# Allelic dimorphism of *Plasmodium vivax gam-1 *in the Indian subcontinent

**DOI:** 10.1186/1475-2875-5-90

**Published:** 2006-10-24

**Authors:** Surendra K Prajapati, Anju Verma, Tridibes Adak, Rajpal S Yadav, Ashwini Kumar, Alex Eapen, Manoj K Das, Neeru Singh, Surya K Sharma, Moshahid A Rizvi, Aditya P Dash, Hema Joshi

**Affiliations:** 1 National Institute of Malaria Research (ICMR), 22-Sham Nath Marg, Delhi, India; 2 National Institute of Malaria Research (Field Unit Nadiad), Gujarat, India; 3 National Institute of Malaria Research (Field Unit Goa), Goa, India; 4 National Institute of Malaria Research (Field Unit Chennai), Tamil Nadu, India; 5 National Institute of Malaria Research (Field Unit Car Nicobar), Andaman & Nicobar Island, India; 6 National Institute of Malaria Research (Field Unit Jabalpur), Madhya Pradesh, India; 7 National Institute of Malaria Research (Field Unit Rourkela), Orissa, India; 8 Department of Biosciences, Jamia Millia Islamia University, Delhi, India

## Abstract

**Background:**

Genetic polymorphism is an inevitable component of a complex organism especially in multistage infectious organisms such as malaria parasites. Understanding the population genetic structure of the parasites would provide valuable information for effective malaria control strategies. Recently, the development of molecular tools like PCR has made analysis of field samples possible and easier and research on *Plasmodium vivax *has also been strengthened. Not many reports are available on the genetic polymorphism of *P. vivax *from the Indian sub-continent. This study evaluates the extent of diversity in field isolates of India with respect to *Pvgam-1*.

**Methods:**

A study was designed to assess the diversity of *Pvgam-1 *among field isolates from India, using a nested PCR assay. Field isolates were collected from different regions of the country and the observed variability was confirmed by sequencing data.

**Results:**

Both Belem and Chesson type alleles were present either exclusively or in mixed form among isolates of all 10 study sites. The Belem type allele was predominant, occurring in 67% of isolates. The proportion of isolates showing the mixed form (both Belem and Chesson type alleles occurring together in the same isolate) was about 13 overall (up to 38.5% in some isolates). Sequencing of the PCR-amplified Belem and Chesson type alleles confirmed the PCR results. Among the 10 study sequences, 11 polymorphic sites and four singleton variations were observed. All the nucleotide substitutions were non-synonymous.

**Conclusion:**

Study shows limited diversity of *Pvgam-1 *marker in Indian isolates with well representation of both Belem and Chesson type alleles.

## Background

*Plasmodium vivax *is the most widespread human malaria parasite and is responsible for 70–80 million cases annually [1]. In India, it is the most prevalent of human malaria parasites, contributing 50–55% cases of malaria each year. *P. vivax *infections are rarely fatal but impact heavily on personal health placing a considerable economic burden upon individuals and communities [2].

Malaria eradication programmes are facing a formidable challenge due to the spread of drug resistance and the complex population genetic structure of malaria parasites. Genetic polymorphism is an inevitable component of a complex organism, especially in multistage infectious organisms such as malaria parasites. Understanding the genetic population structure of the parasites may provide valuable information for effective malaria control strategies. There have been few studies on genetic polymorphism in *P. vivax*, first because more work has been done on the more virulent human malaria parasite, *Plasmodium falciparum*, and secondly because of the non-availability of continuous *in-vitro *cultures for *P. vivax*. Recently, the development of molecular tools, such as PCR, has made analysis of field samples possible and easier, thus strengthening research on *P. vivax*. The availability of the *P. vivax *genome sequence in the public domain has further facilitated this work.

*P. vivax *sub-populations have been reported between temperate zone type (Belem strain) and tropical zone type (Chesson strain) on the basis of different markers such as 18 S SSU rRNA [3], relapse pattern [4], and *Pvcsp *gene [5]. Markers used for exploring population genetic structure of *P. vivax *are limited, namely the *Pvmsp-1 *[6], *Pvmsp*-3α [7], *Pvcsp *[5], *Pvdbp *[8], *Pvama1 *[9], *Pvgam-1 *[10] and microsatellite markers [11]. *Pvgam-1 *is expressed during the gametocyte stage and an antibody raised against the corresponding antigen has been reported to completely block the development of parasites in the mosquito midgut and is reported as potential transmission blocking vaccine candidate [12]. Polymorphism in the C-terminal part of the gene was reported which is due to deletion of 33, 57 & 84 bp guanine-rich motif and is not covered by epitopic region of the PvGAM-1 antigen [12]. However, the validation *of Pvgam-1 *as a genetic marker has been limited so far [13,14]. This paper reports the prevalence of Chesson and Belem type alleles in field isolates from India, on the basis of PCR genotyping of *Pvgam-1 *in different epidemiological regions.

## Materials and methods

### Study sites

**Figure 1:**
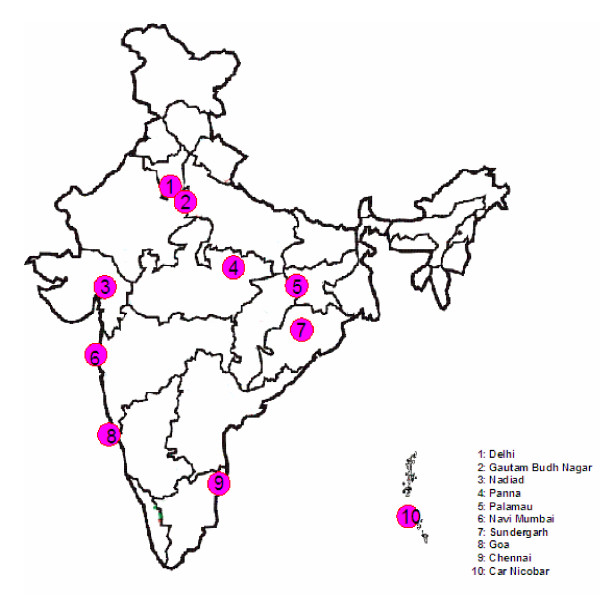
Map of India showing study sites

A total of 252 field isolates were collected from 10 different geographical regions where *P. vivax *is prevalent, including coastal, mainland and Island regions of India (Figure 1). Delhi, Gautam Budh Nagar (Uttar Pradesh), Palamau (Jharkhand), Panna (Madhya Pradesh) and Nadiad (Gujarat) are located in northern region of the country and are under the influence of *An. culicifacies *vector. Transmission is mainly in post-monsoon months. Both species of Plasmodia are present but predominance is different. Except Delhi, in all other study sites, samples were collected during malaria outbreaks. Sites in Gautam Budh Nagar and Nadiad were in rural settings while Panna and Palamau in tribal dominated forested ecotype. Sundergarh district (Orissa) is hyper endemic for malaria with more than 90% of the total malaria cases being that of *P. falciparum *and *P. vivax *poorly reported. It is tribal dominated area under the influence of highly anthropophagic vector *An. fluviatilis*. Chennai (Tamil Nadu), Goa and Navi Mumbai (Maharashtra) are coastal areas with predominance of *P. vivax*. Chennai has a problem of urban malaria with *An. stephensi *as a major vector, while in Goa and Navi Mumbai both *An. stephensi *&*An. culicifacies *are responsible for malaria transmissions. Car Nicobar island in Bay of Bengal is endemic for malaria with perennial transmissions. Both the species i.e., *P. vivax *and *P. falciparum *are prevalent throughout the year and *An. sundaicus *is the vector.

Blood samples were collected during spot surveys or from patients attending a clinic for malaria diagnosis at headquarters in Delhi or at field units, between 2000 and 2004. Microscopically diagnosed *P. vivax*-positive blood was spotted on autoclaved Whatman filter paper (3 mm) strips and dried blood spots were stored at 4°C. Blood smears were stained with JSB stain [15] and examined at a 1,000 × magnification. The study protocol was approved by the ethics committee of the National Institute of Malaria Research, and all blood spots were collected with the consent of the patients.

### Genomic DNA extraction and PCR amplification

Genomic DNA was extracted from three punches of Whatman filter paper with blood spots using QIAamp mini DNA kit (Cat. No.51306, Qiagen, Germany). DNA was eluted in 100 μl sterile triple distilled water. Genomic DNA was analysed using species-specific primers of Tirasophon [16] to confirm the *P. vivax *positivity in the samples. Only isolates having confirmed *P. vivax *infection by PCR were included in the study. All amplification reactions were carried out in a final volume of 20 μl, which included 1 μl template genomic DNA in primary PCR and 0.5 μl of primary product as template in nested PCR. PCR primers and the protocols used for amplification of *Pvgam-1 *were those of Snewin *et al *[10].

### Sequencing of PCR products

PCR products were purified from agarose gel by using gel extraction kit (QIAquick, Cat. No. 28706). Sequencing was done by cycle sequencing method using Big Dye terminator method with ready to use kit (3.1 version, Perkin Elmer Corp) and run on the ABI Prism 310 automated DNA sequencer. Each sample was sequenced by forward and reverse primers to confirm deletion/point mutations in the study fragment of *Pvgam-1*. Sequences were analysed using DNASTAR (Lasergene, USA) software. All DNA sequences were aligned using ClustalW method.

## Results

**Figure 2:**
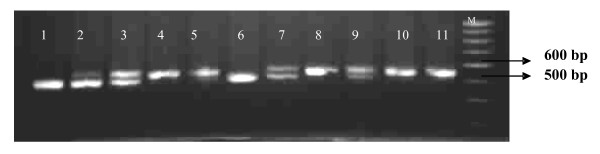
Agarose Gel electrophoretogramme showing allelic variations of *Pvgam-1*. Belem type allele in lane 4, 5, 8, 10 & 11, Chesson type allele in lane 1 & 6 and both alleles in lane nos. 2, 3, 7 & 9. A 100 bp DNA ladder is used as a marker in lane 12

All the 252 isolates showed amplification and observed fragments were 550 bp (Belem) and 520 bp (Chesson) type alleles (Figure 2). These fragments were observed exclusively as well as in combination with each other among the isolates. Table 1 gives the data obtained on the distribution of both Belem and Chesson type alleles among the isolates of different regions. Data revealed that Belem type allele (550 bp) was the predominant allele and exclusively it has occurred in 41.5% to 95.0% of the isolates in different study sites. Proportion of isolates showing Chesson type allele exclusively was upto 61.5%. A good proportion of isolates (13.09 %) showed mixed infection of both the alleles and their proportion ranged in different sites between zero to 38.5% (Table 1).

**Table 1:** Distribution of *Pvgam-1 *alleles in Indian populations

**Study Sites**	**Number of samples tested**	**Observed Allelic Composition of the isolates**		
		
		**Belem (550 bp)**	**Chesson (520 bp)**	**Belem + Chesson**
Delhi	53	22 (41.5%)	16 (30.1%)	15 (28.3%)
Gautam Budh Nagar	8	6 (75%)	2 (25%)	0
Nadiad	50	37 (74%)	4 (8%)	9 (18%)
Navi Mumbai	8	6 (75%)	2 (25%)	0
Goa	36	24 (66.7%)	11 (30.5%)	1 (2.7%)
Chennai	35	31 (88.5%)	2 (5.7%)	2 (5.7%)
Panna	18	14 (77.8%)	4 (22.2%)	0
Palamu	11	10 (91%)	0	1 (9%)
Car Nicobar	20	19 (95%)	1 (5%)	0
Sundergarh	13	0	8 (61.5%)	5 (38.5%)
**Total**	**252**	**169 (67.0%)**	**50(19.8%)**	**33(13.09%)**

Our results are different to earlier observations made among Korean and Thai isolates [13,14]. Korean isolates had shown only Belem type allele while among Thai isolates Chesson type was the predominant allele (92%). Further, Imwong et al [13] observed PCR associated artifacts thus suggested *Pvgam-1 *not to be a dependable marker. In the present study, PCR associated artifacts were not observed in any of the isolates. *Pvgam-1 *is highly GC rich and GC rich regions are reported to be highly prone to illegitimate recombinations thus generating PCR artifacts [17].

**Figure 3:**
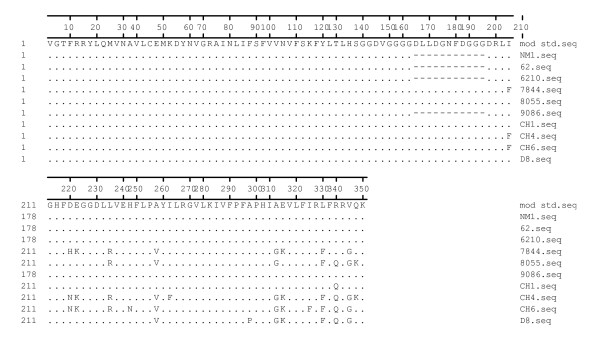
*Pvgam-1 *protein alignments showing amino acid variations.

PCR product from 10 randomly selected isolates (Chennai-Ch 1,4,6 & 62; Delhi-D8; Navi Mumbai- NM1; Goa-6210,7844,8055,9086) were sequenced. Sequencing of the PCR amplified products confirmed the size variations. Alignment of sequences with the reference sequence (Acc. No. X84734) showed good identity and an identity of 85 to 100% was observed between the study isolates. In all the four Chesson type isolates (NM1, 62, 6210, 9086) complete deletion of 33 bp segment was confirmed by sequence alignment. In a total of 10 sequences analyzed in this study, 11 polymorphic sites and 4 singleton variations were observed (Figure 3). Further analysis revealed that polymorphic sites were present only in Belem type allele and not in Chesson type. In Belem type allele similar sequence variations were observed in the isolates of Chennai, Delhi and Goa. All the nucleotide substitutions were non-synonymous. Sequences have been submitted to GenBank vide accession numbers DQ673860-69.

## Discussion

Studies on genetic structure of *P. vivax *is limited mainly because of non-availability of suitable polymorphic markers. Snewin et al [10], added *gam-1 *to the list of available *P. vivax *markers namely *csp, msp-1, msp-3α, dbp*, *ama-1 *by describing a polymorphic region in the *gam-1 *gene (encoding a *P. vivax *transmission blocking candidate antigen) and reported four variants based on nucleotide sequence deletions among 10 Sri Lankan isolates. However, later monomorphic nature of *gam-1 *was observed among Korean isolates [14]. On the other hand, among Thai isolates, both Belem and Chesson type alleles were observed with predominance (92%) of Chesson type allele (520 bp) [13].

Our observations of presence of both Belem and Chesson strains of *P. vivax *among Indian field isolates has been similar to observations of another study reporting presence of the both the strains temperate (Belem) and tropical (Chesson) in Indian population based on the relapsing pattern of vivax infection among the patient [4]. Though the present study could not correlate molecular markers with clinical findings, but predominance of both Belem and Chesson type allele was observed in all the study sites and in isolates collected during spot surveys from malaria endemic as well as during outbreaks.

The polymorphic region of *Pvgam-1 *analysed in this study is known not to be covering the epitopic region responsible for transmission-blocking activity [10]. However, the highly polymorphic nature of the C-terminal region of the fragment in the Belem type allele (11 sites out of 15 showing polymorphism) led to speculation that this region may be under host immune pressure and may also be involved in immunogenicity. In Chesson type alleles, no variation was observed in this region and the deletion of 33 bp repeats may be disrupting the peptide sequence required for antigenicity. This is supported by the conserved nature of this region in all four Chesson type allele (NM1, 62, 6210, 9086) sequenced in this study. Further functional studies are needed to prove whether or not this variable region is involved in immunogenicity.

The highly polymorphic nature of Indian *P. vivax *field isolates had previously been recognized by the relapse pattern [4], drug susceptibility [18] and enzymes polymorphism [19,20]. Based on epidemiological features, Adak et al [4] suggested the existence of polymorphic *P. vivax *population in Delhi region of India and populations were characterized by three types of incubation periods following primary attack of malaria. Short term relapsing strains (Chesson) showed susceptibility to Primaquine and Bulaquine (anti-relapse drugs) while long term relapsing strains (temperate or St. Elizabeth) were not susceptible to these drugs [18].

Studies using biochemical and molecular markers have shown polymorphic forms of Glucose phosphate isomerase (6 alleles), glutamate dehydrogenase (7 alleles) and adenosine deaminase enzymes (5 alleles) [19] as well as size & sequence variations in *msp-3α, csp, gam-1 *[20]. A recent study using *csp, msp-1 *and *msp-3α *in Kolkatta, eastern region, have also reported highly polymorphic nature of Indian *P. vivax *isolates [21].

The present study is first to reveal dimorphic nature of *gam-1 *in the field isolates studied from different parts of India. *Pvgam-1 *marker having two alleles with good frequency distribution among the isolates increases the probability of identifying mixed infections. Studies related to single nucleotide polymorphism, house keeping genes, microsatellite markers as well as drug resistance associated mutations, identification of single clone infection is a prerequisite. Thus, present study suggested *Pvgam-1 *marker could be a useful marker for the identification of multiple infections of different genotypes in addition to *Pvmsp-1, Pvcsp *&*Pvmsp-3α*. Advantage of *Pvgam-1 *marker is that its analysis is performed by means of simple PCR assays and does not require sequencing, hybridization or RFLP analysis as do other markers genes such as *csp *or *msp-3α *locus.

## Conclusion

Data obtained in this study shows limited diversity of *Pvgam-1 *marker in Indian isolates with well representation of both Belem and Chesson type alleles.

## Authors' contributions

SKP: Molecular analysis, PCR work

AV: PCR analysis and sequencing

TA, AE, AK, MKD, SKS, RSY, NS-Epidemiological surveys in different study sites.

MAR: Guidance

APD: Overall Co-ordination and guidance

HJ: Designing, Planning and Co-ordination of the study
